# “Ready-to-use” two-week home exercise program targeting depressive symptoms: pilot study

**DOI:** 10.3389/fpsyt.2023.1202955

**Published:** 2023-09-22

**Authors:** Dasha A. Sandra, Jay A. Olson, Benjamin Pageaux, Mathieu Roy

**Affiliations:** ^1^Department of Psychology, McGill University, Montreal, QC, Canada; ^2^Centre de Recherche de l’Institut Universitaire de Gériatrie de Montréal (CRIUGM), Montreal, QC, Canada; ^3^École de Kinésiologie et des Sciences de l’Activite Physique (EKSAP), Faculté de Médecine, Université de Montréal, Montreal, QC, Canada; ^4^Centre Interdisciplinaire de Recherche sur le Cerveau et l’Apprentissage (CIRCA), Montreal, QC, Canada

**Keywords:** exercise, interventions, depression, university students, COVID-19

## Abstract

Exercise is an evidence-based treatment for depressive symptoms, yet it often requires specialised knowledge, equipment, or professional supervision. Lay people in certain contexts, for example in remote locations or under pandemic restrictions, often lack these resources and thus cannot use exercise to manage their depressive symptoms. We developed a two-week home exercise program that bypasses these barriers and tested it in university students during pandemic restrictions. In an online study, we recruited 49 participants to complete a week of baseline symptom monitoring then follow the exercise program for 2 weeks (6 sessions) at home. The exercise program involved aerobic and resistance training; each session lasted approximately 45 min. After 2 weeks of the intervention, participants reported lower depressive (standardised β = −0.71 [−1.05, −0.38]) and anxiety (β = −0.87 [−1.19, −0.55]) symptoms. Although we cannot make causal conclusions, our results suggest that the brief home exercise program may have potential to reduce depressive symptoms in young adults.

## 1. Introduction

Depression is the leading cause of disability, affecting 265 million people worldwide (1). Around 21% of Americans suffer from depression in a given year (2), and this number increased to 33% during the COVID-19 pandemic (3). Physical exercise is one of the widely accepted effective interventions to treat mild to moderate depressive symptoms (4). It may be as effective as antidepressants (5).

The effectiveness of exercise in reducing depressive symptoms relies on the combination of several factors such as the type of exercise, its intensity, and the general context in which it is completed. Aerobic exercise, which targets cardiovascular and respiratory systems, improves depressive symptoms; combining it with resistance training, which targets neuromuscular function, may further increase its effectiveness (6). Vigorous exercise, which requires more energy and physical effort to complete, is also more effective than less intense activities, such as stretching or walking (6). While type and intensity increase the effectiveness of exercise itself, the social context also contributes to compliance. For instance, exercising alone and in groups together increases compliance overall more than exercising either only alone or only in groups (7). Finding the optimal combination of factors to reduce depressive symptoms often requires specialised knowledge and access to particular types of equipment. However, access to these can be scarce in areas with few kinesiologists, in regions affected by public health crises such as the COVID-19 pandemic, or for predominantly low-income populations (e.g., university students). Indeed, a lack of access to specialised knowledge and equipment are common barriers to using exercise to for depressive symptoms management (8). A possible solution, then, would be to develop a “ready-to-use,” free, and safe intervention with all the necessary information, such as specific exercises, as well as their frequencies and duration. Such an intervention would be particularly useful if it could be completed at home without specialised equipment. Few interventions of this type exist, despite many remote exercise programs having been developed for various populations. To address this need, we developed a standardised exercise program and tested it over 2 weeks on university students with depressive symptoms. We tested the program during the peak of the COVID-19 pandemic lockdowns (i.e., closed gyms, overnight curfew), during which such an intervention would be most useful. The program requires no special equipment, can be quickly explained, and needs no supervision. A potential drawback of generic programs, however, is the lack of personal attention and individualisation; indeed, presenting treatments as personalised may enhance their effectiveness in some settings (9). Expecting personalisation may be especially relevant for exercise, where there is often considerable individual variation. We therefore also tested whether presenting the intervention as personalised (without actually adjusting any intervention elements) could remedy the issue. We hypothesised that participants in both groups would report a reduction in depressive and anxiety symptoms, but especially so when the intervention was presented as having been personalised to them.

## 2. Materials and methods

### 2.1. Participants

We focused on university students due to their high levels of reported mental health problems (10). Using a pre-screening, we recruited 54 students who scored 10 or higher on the Beck Depression Inventory-II, reported no prior diagnosis of depression or other psychiatric disorders, were not taking any psychoactive medications, and were not actively exercising (defined as exercising at least 3 times per week). Participants enrolled in the study between February and June 2021 in Montreal, Canada, while most social and physical activities were restricted and classes were online due to the COVID-19 pandemic. We excluded 5 participants from the final sample given that they quit the study before being assigned to the condition. The final sample included 49 participants. They were on average 23.1 years old (*SD* = 4.0) and predominantly female (80% women). The study was approved by the McGill University Research Ethics Board (#20–11-007).

### 2.2. Intervention

Participants followed a 14-d intervention program requiring no prior training or specialised knowledge about physical exercise. The program was based on the literature and included both resistance and aerobic exercises to maximise the reduction of depressive symptoms (6, 11); it was also designed to be feasible to complete at home. It began with a warm-up of 5 min of walking or climbing stairs. Next, it included resistance training with 4 sets of 15 repetitions of each of the following exercises: lunges, push-ups, squats, makeshift rowing with a towel for resistance, and back bridges. Finally, the program suggested 4 sets of 30 s of front plank and side planks on each side. The program also included aerobic exercise involving 15 min of brisk walking outside (see the full program in the Supplementary Material). Participants were free to choose the order of the exercises and when to complete them during the day but were asked to do all exercises in one session each time. Each session lasted between 45 and 50 min.

### 2.3. Procedure

The study took place online. During the first virtual meeting, participants gave informed consent, completed the Beck Depression Inventory-II (BDI-II) and the Hospital Anxiety and Depression Scale (HADS), and then entered a waitlist. One week later, they attended another virtual meeting, during which they first completed the same questionnaires and then received the exercise intervention. The experimenter walked the participants through each step of the exercise program and encouraged them to follow it three times per week for the next 14 days (6 sessions). Participants were offered a bonus of $20 in addition to the study compensation of $30 if they reported having completed more than half of the sessions. If participants reported any suicidal thoughts (a score above 0 on the BDI-II suicide question), the experimenter discussed it with the participants and presented them with mental health resources available on campus.

To test whether the expectation of receiving a personalised intervention affected depressive symptoms, we randomised participants to a ‘personalised’ or ‘control’ group. The participants completed a series of questionnaires about their health history of depressive symptoms and personality traits. In the ‘personalised’ group, the experimenter pretended to tailor the intervention based on these individual questionnaire answers. Specifically, the experimenter described an algorithm developed in collaboration between McGill University and the UK Biobank to improve the treatment of depression. Participants in the experimental group then received the program presented as the algorithm’s choice given their individual responses. Those in the ‘standard’ group completed the same measures and received the same program, but it was instead described as a professionally developed standard exercise program intervention for depression.

### 2.4. Measures

#### 2.4.1. Beck Depression Inventory-II (BDI-II)

The BDI-II assesses the severity of depressive symptoms over the past 2 weeks (12). The questionnaire has 21 items with scores ranging from 0 to 63, with 14 being the cut-off for mild clinical depression. Each item measures a specific area (e.g., sadness) and has answers varying from 0 to 3, for example, “I do not feel sad” (0 points) to “I am so sad or unhappy that I cannot stand it” (3 points). The scale’s internal consistency of the sample at baseline assessment was excellent (Cronbach’s *𝛼* = .92).

#### 2.4.2. Hospital Anxiety and Depression Scale (HADS)

The HADS measures depression and anxiety over the past week (13) and is widely used for a variety of clinical populations. The questionnaire includes 14 items: 7 for anxiety and 7 for depression. Participants answer items such as “I feel tense or ‘wound up’” on a scale from 0 (Not at all) to 3 (Most of the time). Anxiety and depression are measured separately with scores ranging from 0 to 21 and a clinical cut-off of 8 for both. Internal reliability for subscales was acceptable or high at baseline assessment (HADS-D *𝛼* = .78, HADS-A *𝛼* = .87).

### 2.5. Analysis plan

We separately tested the effectiveness of the intervention to reduce symptoms of depression and anxiety in an intention-to-treat analysis. We ran two separate mixed-effects models on the outcomes (depressive and anxiety symptoms) given the time (pre- or post-intervention), the condition (control or personalised), and the interaction between the two variables, with a random intercept for each participant. We tested the main effects on each dependent variable (the effect of the intervention overall) and the interaction (the effect of expectation on treatment effectiveness), using a Type I error rate of .05 and directional tests (*nlme* package in R, *version 4.3.1*). To account for missing values, we used multiple imputation with predictive mean matching (14) from the *mice* package in R. We predicted that depressive symptoms would be lower post-intervention, and especially lower in the ‘personalised’ group when compared to control. The design, sample size, and analyses testing the effects of expectation on treatment outcomes (interaction effect) were pre-registered online, but we stopped data collection early.^1^

## 3. Results

### 3.1. General effectiveness of exercise

Participants reported clinically meaningful reductions in depressive symptoms after completing the intervention. Participants improved by 10.8 points, or 40%, in their depression scores on the BDI, which ranges from 0 to 63 (standardised β_BDI-II_ = −0.71 [−1.05, −0.38], *p* <  .001). They reported similar reductions on the depression scale of the HADS, which ranges from 0 to 21, with decreases of 2.6 points (β_HADS-D_ = −0.63 [−1.03, −0.23], *p* = .003). Similarly, participants decreased on their anxiety symptoms by 3.8 points after the intervention (β_HADS-A_ = −0.87 [−1.19, −0.55], *p* <  .001). All reductions were clinically significant, given the minimal clinically important difference (MCID) threshold of between 1.7 and 2.5 raw points for each subscale of the HADS (15, 16), and a MCID threshold of 17.5% reduction for the BDI-II. From the final sample, 38 participants (78%) reached the MCID on their BDI scores, and 28 participants (57%) on their HADS-D scores.

**Figure 1:**
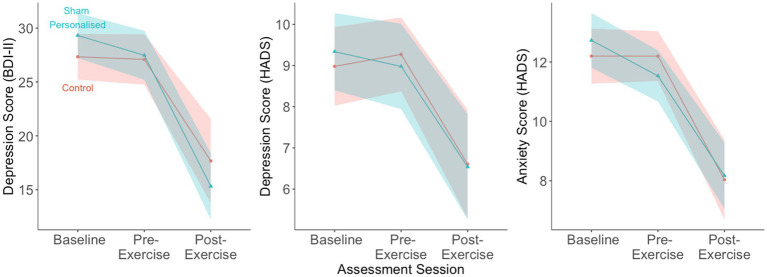
Participants (*N* = 49) reported large reductions in depressive and anxiety symptoms after following the exercise intervention for two weeks. The reductions were similar across the two groups. Dots show means, shaded regions show 95% confidence intervals.

During the first week, participants showed no reductions in symptoms on any measures (β_BDI-II_ = −0.02 [−0.20, 0.16], β_HADS-D_ = 0.07 [−0.22, 0.37], β_HADS-A_ = 0.00 [−0.23, 0.23]), suggesting that simply being enrolled in a study or answering questionnaires did not lead to symptom reduction (Figure 1).

### 3.2. Personalisation manipulation

Participants reported similar reductions in anxiety and depression in both groups (interaction β_BDI-II_ = −0.21 [−0.68, 0.26], β_HADS-D_ = 0.05 [−0.50, 0.61], β_HADS-A_ = 0.17 [−0.28, 0.62]) (Table 1).

## 4. Discussion

We tested whether a two-week home-based and unsupervised exercise intervention could improve depressive and anxiety symptoms for university students during a COVID-19 lockdown. Participants reported large reductions in depressive and anxiety symptoms after following the intervention for only 2 weeks. Believing the intervention to be personalised to one’s symptoms and health characteristics did not affect its efficacy and led to similar improvements in depression and anxiety.

Our intervention has several strengths. The program requires no supervision, specialised equipment, or prior knowledge of exercise, and includes familiar exercises. Our program can allow individuals to quickly begin the intervention without the need for training or supervision, overcoming a common barrier to engaging in exercise (8). Indeed, lacking concrete knowledge of how to begin a habit is a common barrier to uptake.

Further, participants in the study showed large improvements in depressive symptoms after only 2 weeks. This is faster than most existing interventions in the literature, which last 10 weeks on average (17). Our study demonstrated that exercising may potentially rapidly decrease depressive symptoms; however, a longer intervention is likely needed to maintain the initial gains in improvement and implement regular physical activity in daily life. Nevertheless, a brief intervention could be helpful for individuals suffering from milder depressive symptoms, as exercise is most effective for mild to moderate depression (11). Future studies may also test the intervention as a tool to prevent relapse for those who are currently in remission but are dealing with external stressors.

The remote nature of the exercise program may be especially useful for people living in rural areas, far from physical activity centers, or those who exercise less. Given that physical activity is generally declining around the world (18) and that increases in sedentary behaviour may be associated with a higher risk of depression (19), our simple exercise intervention may present a potential buffer. Future studies could explore whether the level of baseline sedentary lifestyle moderates the effectiveness of remote exercise interventions. Finally, the program was equally effective when presented as standard or personalised to individual characteristics. We expected that the typical approach of tailoring exercise to an individual’s needs may increase expectations of effectiveness. Here, we present tentative evidence that framing related to personalisation may play a smaller role in remotely delivered exercise programs.

The largest limitation in our study is our inability to make causal conclusions due to the lack of a no-intervention control group. Although both groups showed reductions in depression and anxiety, and these reductions began after the baseline monitoring, it is unclear to what extent these may have been caused by demand characteristics, positive expectations, reductions in the stressors associated with the university semester, or simply the passing of time. However, the effect sizes of such a brief intervention in a pilot study are sufficiently promising to warrant further study against a no-intervention control group. For example, studies could test the intervention against an active control such as stretching, as well as in various populations with different levels of baseline physical activity. With rates of depression increasing and those of physical activity decreasing around the world, accessible exercise programs may be a sustainable option to mitigate both.

**Table 1:** Regression model coefficients for confirmatory findings.

Outcome	Predictor	*β*	CI	SE	*T*	*df*	*p*
Depression scores (BDI-II)	(Intercept)	0.42		0.21			
	Condition	0.05		0.27			
	**Time**	**−0.71**	**−1.05, −0.38**	**0.17**	**−4.3**	**47**	**< .001**
	Interaction	−0.21	−0.68, 0.26	0.23	−0.89	47	.38
Depression scores (HADS-D)	(Intercept)	0.25		0.19			
	Condition	0.10		0.27			
	**Time**	**−0.63**	**−1.03, −0.23**	**0.20**	**−3.20**	**47**	**.003**
	Interaction	0.05	−0.50, 0.61	0.28	0.19	47	.85
Anxiety scores (HADS-A)	(Intercept)	0.39		0.20			
	Condition	0.01		0.28			
	**Time**	**−0.87**	**−1.19, −0.55**	**0.16**	**−5.5**	**47**	**< .001**
	Interaction	0.17	−0.28, 0.62	0.22	0.76	47	.45

Statistically significant results are bolded.

## Data availability statement

The datasets presented in this study can be found in online repositories. The names of the repository/repositories and accession number(s) can be found below: https://osf.io/nfb6w/.

## Footnotes

^1^ https://osf.io/nqzj7
