# Supplementary material for: “Ready-to-use” two-week home exercise program targeting depressive symptoms: pilot study
Source: Front Psychiatry. 2023 Sep 22;14:1202955. doi: 10.3389/fpsyt.2023.1202955 (PMC10563784; doi:10.3389/fpsyt.2023.1202955)
Supplement: Supplementary file 1 [file Table_1.pdf]

INTERVENTION:	TWO-WEEK UNSUPERVISED EXERCISE PROGRAM
**Warm-up**	5 minutes of walking **OR** climbing stairs in a relaxed manner

**Muscle Training**	Number of repetitions **OR** time spent on exercise	Number of sets (number of times you need to complete the repetitions **OR** time spent on exercise)
Lunge	**15 repetitions each side**	**4 sets required**
Plank	**30 seconds**	**4 sets required**
Side plank	**30 seconds each side**	**4 sets required**
Push-up	**15 repetitions**	**4 sets required**
Squat	**15 repetitions**	**4 sets required**
Towel row	**15 repetitions**	**4 sets required**
Back bridge	**15 repetitions**	**4 sets required**

**Cardiovascular Training**	15 minutes of walking outside (should feel warm and slightly out of breath)

Exercise	Instructions for each exercise	Example of exercise
Lunge	Standing up straight, with feet hip-width apart, extend one foot in front of the other, about an arms-length distance apart. Contract abdomen and begin to lower hips until both knees are at about 90 degrees to the ground.<br>**YouTube video explaining exercise:** https://www.youtube.com/watch?v=QOVaHwm-Q6U	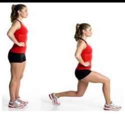
Plank	Place hands on floor, underneath shoulders, keeping arms shoulder-width apart. Place toes on ground, hip-width apart, to create a straight strong line from head to toe.<br>**YouTube video explaining exercise:** https://www.youtube.com/watch?v=u6ZeIKyUM6g	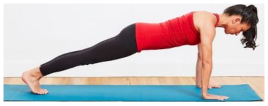
Side plank	Place one hand on floor, aligning shoulder and wrist. Middle finger points forward, and head and heel form a straight alignment by having knees straight and feet stacked.<br>**YouTube video explaining exercise:** https://www.youtube.com/watch?v=XeN4pEZZJNI	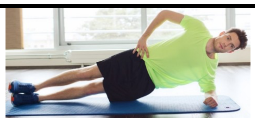
Push-up	Arms shoulder-width apart, place hands on floor, aligned under shoulders. Lower your body by bending the arms while keeping the back flat and head slightly extended, until almost complete flexion. Return to the starting position with alignment.<br>**YouTube video explaining exercise:** https://www.youtube.com/watch?v=bt5b9x9N0KU	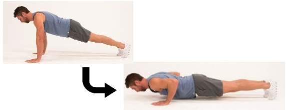
Squat	Place feet a little wider than shoulder-width and toes slightly outward. Bend hips backwards, while knees stay above the feet always. Unbend by pushing hips up without hyper-tension of the knees.<br>**YouTube video explaining exercise:** https://www.youtube.com/watch?v=aclHkVaku9U	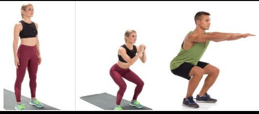
Towel row	Standing up straight, with feet shoulder-width apart, strongly stretch towel outward, with elbows completely unbent. Bring towel close to chest while keeping the strong pull (as if to tear the towel) then return by unbending elbows, always keeping the tension/stretch on the towel.<br>**YouTube video explaining exercise:** https://www.youtube.com/watch?v=lPZufNTFFHQ	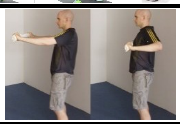
Back bridge	Lying down on your back, with hands on sides, knees bent, and feet at shoulder-width apart. Make sure knees are bent properly (if you are able to reach your heels with your hands, you are positioned well). Push slowly on heels to lift hips off the floor while keeping your back straight. Return slowly to the starting position.<br>**YouTube video explaining exercise:** https://www.youtube.com/watch?v=wPM8icPu6H8	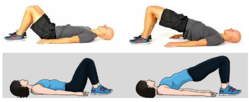

Exercise schedule

**Week 1**	Sunday	Monday	Tuesday ✓	Wednesday	Thursday ✓	Friday	Saturday ✓
**Week 2**	Sunday	Monday	Tuesday ✓	Wednesday	Thursday ✓	Friday	Saturday ✓
